# Low-Level Lead Exposure Increases Systolic Arterial Pressure and Endothelium-Derived Vasodilator Factors in Rat Aortas

**DOI:** 10.1371/journal.pone.0017117

**Published:** 2011-02-25

**Authors:** Jonaina Fiorim, Eduardo Hertel Ribeiro, Edna A. Silveira, Alessandra S. Padilha, Marcos Vinícius A. Vescovi, Honério C. de Jesus, Ivanita Stefanon, Mercedes Salaices, Dalton V. Vassallo

**Affiliations:** 1 Department of Physiological Sciences, Federal University of Espirito Santo, Vitoria, Espírito Santo, Brazil; 2 Health Science Center of Vitória- EMESCAM, Vitoria, Espírito Santo, Brazil; 3 Departamento de Farmacología, Universidad Autónoma de Madrid, Madrid, Spain; University of Illinois at Chicago, United States of America

## Abstract

Chronic lead exposure induces hypertension and alters endothelial function. However, treatment with low lead concentrations was not yet explored. We analyzed the effects of 7 day exposure to low lead concentrations on endothelium-dependent responses. Wistar rats were treated with lead (1st dose 4 µg/100 g, subsequent dose 0.05 µg/100 g, i.m. to cover daily loss) or vehicle; blood levels attained at the end of treatment were 9.98 µg/dL. Lead treatment had the following effects: increase in systolic blood pressure (SBP); reduction of contractile response to phenylephrine (1 nM–100 µM) of aortic rings; unaffected relaxation induced by acetylcholine (0.1 nM–300 µM) or sodium nitroprusside (0.01 nM–0.3 µM). Endothelium removal, *N*^G^-nitro-L-arginine methyl ester (100 µM) and tetraethylammonium (2 mM) increased the response to phenylephrine in treated rats more than in untreated rats. Aminoguanidine (50 µM) increased but losartan (10 µM) and enalapril (10 µM) reduced the response to phenylephrine in treated rats. Lead treatment also increased aortic Na^+^/K^+^-ATPase functional activity, plasma angiotensin-converting enzyme (ACE) activity, protein expression of the Na^+^/K^+^-ATPase alpha-1 subunit, phosphorylated endothelial nitric oxide synthase (p-eNOS), and inducible nitric oxide synthase (iNOS). Our results suggest that on initial stages of lead exposure, increased SBP is caused by the increase in plasma ACE activity. This effect is accompanied by increased p-eNOS, iNOS protein expression and Na^+^/K^+^-ATPase functional activity. These factors might be a compensatory mechanism to the increase in SBP.

## Introduction

Lead is considered an environmental pollutant of high risk to public health [1], [2].This metal is extensively used by the industrial sector, thereby contributing to its wide environmental distribution. Usually, plasma lead levels in humans result from exposure to exogenous sources [3]. Occupational exposure occurs during the manufacture of ammunition, batteries, solder, ceramic glazes, plastics and sheet lead [4]. Lead affects the central nervous system [3] and the renal system [5], and population studies have demonstrated an association between lead exposure and kidney disease [6], [7]. Recently, more research has focused on the toxic effects of lead on the cardiovascular system and its association with hypertension in humans [8]–[10] and animals [11]–[13]. Several mechanisms have been proposed to cause lead-induced hypertension, such as alterations in calcium exchangeability [14], central sympathetic activity enhancement [15], [12], increases in plasma catecholamines [16], inhibition of the Na^+^/K^+^-ATPase [17], direct activation of smooth muscle protein kinase C [18], increased activity of the renin-angiotensin system [19] and endothelial dysfunction [20], [21].

Endothelial dysfunction is associated with hypertension and with a concomitant increase in morbidity and mortality [22], [23]. Vaziri et al. [21], [22] showed that treatment with 100 ppm of lead in drinking water for 12 weeks induces hypertension and endothelial dysfunction in Sprague-Dawley rats. Several reports have shown that the chronic lead treatment with 100 ppm in drinking water increases vascular reactivity to phenylephrine in aortic rings [24], [25], [13], although other authors have demonstrated a decrease in the vascular reactivity to phenylephrine after lead treatment [26]. It is still unclear whether this altered vascular reactivity can contribute to lead-induced hypertension.

The Agency for Toxic Substances and Disease Registry (ATSDR) recommends that the concentration of lead in the blood is under 60 µg/dL in adults that experience occupational lead exposure [3], [27], [28]; nevertheless, individuals with blood lead concentrations between 31.4 µg/dL and 53.5 µg/dL showed an increase in arterial pressure [6], [9]. Recent experimental studies of lead toxicity demonstrated a concentrations of lead in the blood between 31.8 µg/dL and 58.7 µg/dL [29], [30], [13]; these concentrations are similar to those found in the population of workers exposed to lead; however, little attention has been given to the effects of lead in the blood at concentrations below those found in humans with occupational lead exposure and the effects during the initial stages of lead exposure. Because of the lack of knowledge in these areas, we developed an experimental model of lead exposure in rats that produces blood concentrations below those found in humans with occupational lead exposure. Thus, the aim of this study was to investigate the effects of seven-day treatment with a low concentration of lead acetate on the systolic blood pressure (SBP) and vascular reactivity in aortic rings.

## Materials and Methods

### Animals and treatment

Male Wistar rats (260–300 g) were used for these studies. The care and use of laboratory animals were in accordance with the NIH guidelines, and all experiments were conducted in compliance with the guidelines for biomedical research as stated by the Brazilian Societies of Experimental Biology and were approved by the Institutional Ethics Committee of the Health Science Center of Vitória (CEUA-EMESCAM 004/2007). All rats had free access to water and were fed rat chow *ad libitum*. Rats were divided into two groups: control (vehicle-saline, i.m.) or treated with lead acetate for seven days (1st dose: 4 µg/100 g, subsequent dose: 0.05 µg/100 g, i.m. to cover daily loss). At the end of the treatment, rats were anesthetized with pentobarbital (35 mg/kg, i.p.) and killed by exsanguination. Thoracic aortas were carefully dissected out and connective tissue was removed. For vascular reactivity experiments, the aortas were divided into cylindrical segments 4 mm in length. For analysis of protein expression, some arteries were rapidly frozen in liquid nitrogen and stored at −80°C until analyzed. Blood samples were collected in tubes without EDTA and placed in ice and then centrifuged at 3,500×*g* for 15 min at 4°C. The resulting plasma was stored at −80°C until use to determine ACE activity.

### Blood pressure measurements

Indirect systolic blood pressure was measured at the beginning and at the end of treatment using tail-cuff plethysmography (IITC Life Science, Inc). Conscious rats were restrained for 5–10 min in a warm and quiet room and conditioned to numerous cuff inflation-deflation cycles by a trained operator. Systolic blood pressure was measured, and the mean of three measurements was recorded [13].

### Blood lead level measurements

Blood lead level measurements were determined according to the protocol developed by Korecková-Sysalová [31]. Lead concentrations in samples of whole blood after 7 days of treatment were measured in duplicate by atomic fluorescence spectrometry (model: AAS5 EA with graphite furnace, Carl Zeiss, Germany) at the Centre for Exact Sciences - Chemistry Department (Federal University of Espirito Santo).

### Vascular reactivity measurements

Aortic segments (4 mm in length) were mounted between two parallel wires in 37°C organ baths containing Krebs-Henseleit solution (KHS, in mM: 124 NaCl, 4.6 KCl, 2.5 CaCl_2_, 1.2 MgSO_4_, 1.2 KH_2_PO_4_, 0.01 EDTA, 23 NaHCO_3_) and gassed with 95% O_2_-5% CO_2_ (pH 7.4). Arterial segments were stretched to an optimal resting tension of 1.0 g. Isometric tension was recorded using a force displacement transducer (TSD125C, CA, USA) connected to an acquisition system (MP100A, BIOPAC System, Inc., Santa Barbara, USA).

After a 45 min equilibration period, all aortic rings were initially exposed twice to 75 mM KCl. The first exposure checks their functional integrity, and the second exposure assesses the maximal tension developed. Afterwards, endothelial integrity was tested with acetylcholine (10 µM) in segments previously contracted with phenylephrine (1 µM). A relaxation equal to or greater than 90% was considered demonstrative of the functional integrity of the endothelium. After a 45 min washout, concentration-response curves to phenylephrine were determined. Single curves were performed in each segment. Effects of the nonspecific NOS inhibitor *N*^G^-nitro-L-arginine methyl ester (L-NAME, 100 µM), the inducible NO synthase (iNOS) inhibitor aminoguanidine (50 µM), the potassium (K^+^) channel blocker tetraethylammonium (TEA, 2 mM), the cyclooxygenase inhibitor indomethacin (10 µM), the AT_1_ receptor blocker losartan (10 µM) and the ACE inhibitor enalapril (10 µM) were investigated. These drugs were added to the bath 30 min before performing the phenylephrine concentration-response curves.

The influence of the endothelium on the response to phenylephrine in untreated and treated- lead rats was investigated after its mechanical removal, which was performed by rubbing the lumen with a needle. The absence of endothelium was confirmed by the inability of 10 µM acetylcholine (ACh) to produce relaxation.

In another set of experiments, after the 45 min equilibration period, aortic rings from untreated and lead-treated rats were pre-contracted with phenylephrine (1 µM), and concentration-response curves to acetylcholine (0.1 nM–300 µM) or sodium nitroprusside (0.01 nM–0.3 µM) were determined.

The functional activity of the Na^+−^/K^+^-ATPase in segments from control and lead-treated rats was measured using K^+^-induced relaxation, as described by Webb and Bohr [32] and modified by Rossoni et al. [33]. After a 30-min equilibration period in normal Krebs, preparations were incubated for 30 min in K^+^-free Krebs. The vessels were subsequently pre-contracted with phenylephrine, and once a plateau was attained, the concentration of KCl was increased step-wise (1, 2, 5 and 10 mM), with each step lasting for 2.5 min. After these procedures, preparations were incubated with 100 µM ouabain for 30 min to inhibit sodium pump activity, and the K^+^-induced relaxation curve was repeated.

### Determination of angiotensin-converting enzyme (ACE) activity

The effect of lead treatment on serum angiotensin-converting enzyme (ACE) activity was determined according to the protocol detailed by Oliveira et al. [34]. Briefly, serum (3 µl) was incubated with 40 µl of assay buffer containing 5 mM Hip-His-Leu in 0.4 M sodium borate buffer with 0.9 M NaCl, pH 8.3 for 15 min at 37°C. The reaction was stopped by the addition of 190 µl of 0.34 N NaOH. The product, His-Leu, was measured fluorometrically at an excitation wavelength 365-nm and an emission wavelength of 495-nm using a fluoro-colorimeter (Synergy 2, Biotek). Seventeen microliters of *o*-phthaldialdehyde (20 mg/ml) in methanol was added. To correct for the intrinsic fluorescence of the serum, time zero blanks (T_o_) were prepared by adding serum after NaOH. All assays were performed in triplicate.

### Western blot analyses

Proteins from homogenized arteries (50 µg for eNOS, p-eNOS, iNOS, AT_1_, AT_2_ receptors and 80 µg for Na^+^/K^+^-ATPase alpha-1 and 2 subunits) were separated by 10% SDS-PAGE. Proteins were transferred to nitrocellulose membranes that were incubated with mouse monoclonal antibodies for endothelial nitric oxide synthase (eNOS), phosphorylated endothelial nitric oxide synthase (p-eNOS; 1∶250; Transduction Laboratories, Lexington, UK), inducible nitric oxide synthase (iNOS; 1∶250; Transduction Laboratories, Lexington, UK), Na^+^/K^+^-ATPase alpha-1 subunit (1∶1000; Millipore, San Francisco, USA) or Na^+^/^−^K^+^-ATPase alpha-2 subunit (1∶500; Millipore, San Francisco, USA) or rabbit polyclonal antibodies for AT_1_ or AT_2_ (1∶500; Biotechnology, Santa Cruz, USA). After washing, membranes were incubated with anti-mouse (1∶5000; StressGen, Victoria, Canada) or anti-rabbit (1∶7000; StressGen, Victoria, Canada) antibodies conjugated to horseradish peroxidase. After thorough washing, immunocomplexes were detected using an enhanced horseradish peroxidase/chemiluminescence system (ECL Plus, Amersham International, Little Chalfont, UK) and film (Hyperfilm ECL International). Signals on the immunoblots were quantified with the National Institutes of Health Image V1.56 software. The same membranes were used to determine α-actin expression using a mouse monoclonal antibody to α-actin (1∶5000; Sigma, USA).

### Statistical analyses

All values are expressed as mean ± S.E.M. Contractile responses were expressed as a percentage of the maximal response induced by 75 mM KCl. Relaxation responses to ACh or NPS were expressed as the percentage of relaxation of the maximal contractile response. For each concentration-response curve, the maximal effect (R_max_) and the concentration of agonist that produced 50% of the maximal response (log EC_50_) were calculated using non-linear regression analysis (GraphPad Prism, GraphPad Software, Inc., San Diego, CA). The sensitivities of the agonists were expressed as pD_2_ (−log EC_50_). To compare the effects of endothelium denudation or L-NAME on the contractile responses to phenylephrine, some results were expressed as differences in the area under the concentration response curves (dAUC) for the control and experimental groups. These values indicate whether the magnitude of the effect of either endothelial denudation or L-NAME is different in untreated or lead-treated rats. Relaxation induced by K^+^ was expressed as a percentage of the tone previously obtained with phenylephrine. The curves of relaxation induced by K^+^ were constructed through the analysis of nonlinear regression of the concentration-response curves.

For protein expression, data were expressed as the ratio between signals on the immunoblot corresponding to the protein of interest and α-actin. For protein expression of p-eNOS, data were expressed as the ratio between p-eNOS/eNOS. Results are expressed as mean ± SEM of the number of rats indicated; differences were analyzed using Student's *t*-test or two-way ANOVA followed by a Bonferroni test. *P*<0.05 was considered significant.

### Drugs and reagents

Lead acetate Pb(CH_3_COO)2, l-phenylephrine hydrochloride, L-NAME, enalapril, indomethacin, acetylcholine chloride, sodium pentobarbital, losartan, ouabain, sodium nitropusside, SOD, aminoguanidine and tetraethylammonium were purchased from Sigma-Aldrich (St. Louis, USA). Salts and reagents used were of analytical grade from Sigma-Aldrich and Merck (Darmstadt, Germany).

## Results

No differences in body weight between the two groups were observed before (untreated: 261.1±1.39 g, n = 40; lead-treated: 259±0.98 g, n = 40; P>0.05) or after treatment (untreated: 305.5±2.88 g, n = 40; lead-treated: 310.1±2.68 g, n = 40; P>0.05).

In rats exposed to seven-day lead treatment, the blood lead concentration attained was 9.98 µg/dL±1.70 µg/dL (n = 5). A significant rise in systolic arterial blood pressure was observed seven days after lead exposure (untreated: 121±1.50 mmHg, n = 12; lead treated: 137±2.36 mmHg, n = 12, P<0.05).

### Effects of lead treatment on vascular reactivity

**Figure 1:**
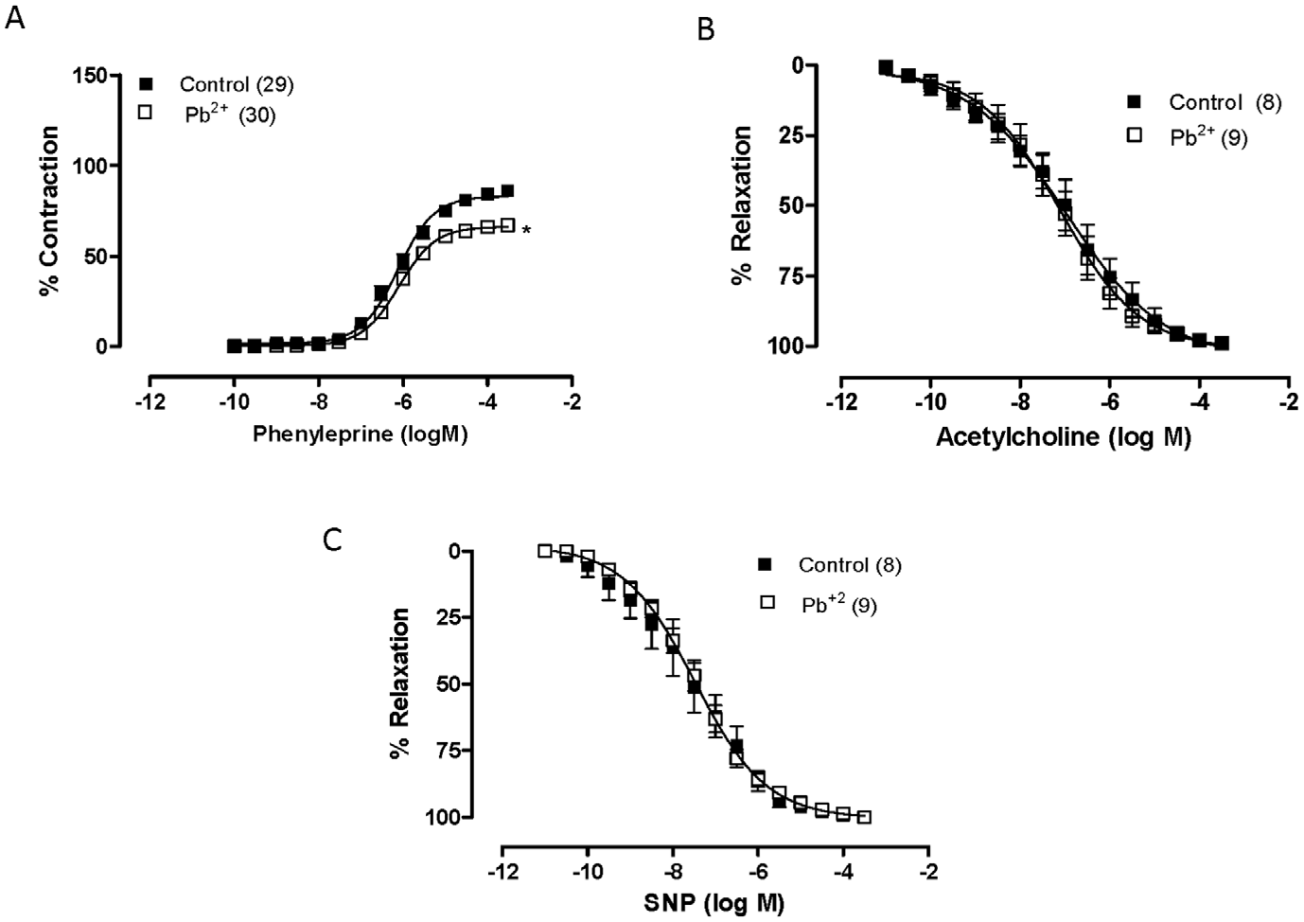
The effects of seven-day exposure to lead acetate on the concentration-response curves to phenylephrine (A), acetylcholine (B) and sodium nitroprusside (NSP; C) treatment in aortic rings. *P<0.05 by Student's *t-*test. Number of animals used is indicated in parentheses.

Lead treatment did not affect the response to KCl (untreated: 3.48±0.08 g, n = 39; lead-treated: 3.52±0.09 g, n = 40; P>0.05), but it decreased the contractile responses induced by phenylephrine in rat aortas (Figure 1 A). It also decreased *R_max_* but not sensibility to phenylephrine (Table 1).

**Table 1:** Parameters of maximal response (R_max_) and sensitivity (pD_2_) of the concentration-response curves to phenylephrine in the aortas from untreated and lead-treated rats, before (E+) and after (E−) endothelial damage and after *N*^G^-nitro-L-arginine methyl ester (L-NAME, 100 µM) and indomethacin (10 µM) incubation.

	Untreated		Lead treated	
	Rmax	pD2	Rmax	pD2
**Control**	81.80±4,46	6.55±0,1	66.15±4,66^#^	6.29±0,06
**E^−^**	150. 4±7,32^#^	7.65±0.14^#^	152.1±10,43^*^	7.37±0.10^*^
**L-NAME**	130.1±2,57^#^	7.69±0.23^#^	148.9±3.22*	7.12±0.05*
**Indomethacin**	85. 64±8.71	6.64±0,08	64.61±5.67	6.39±0.07

**Figure 2:**
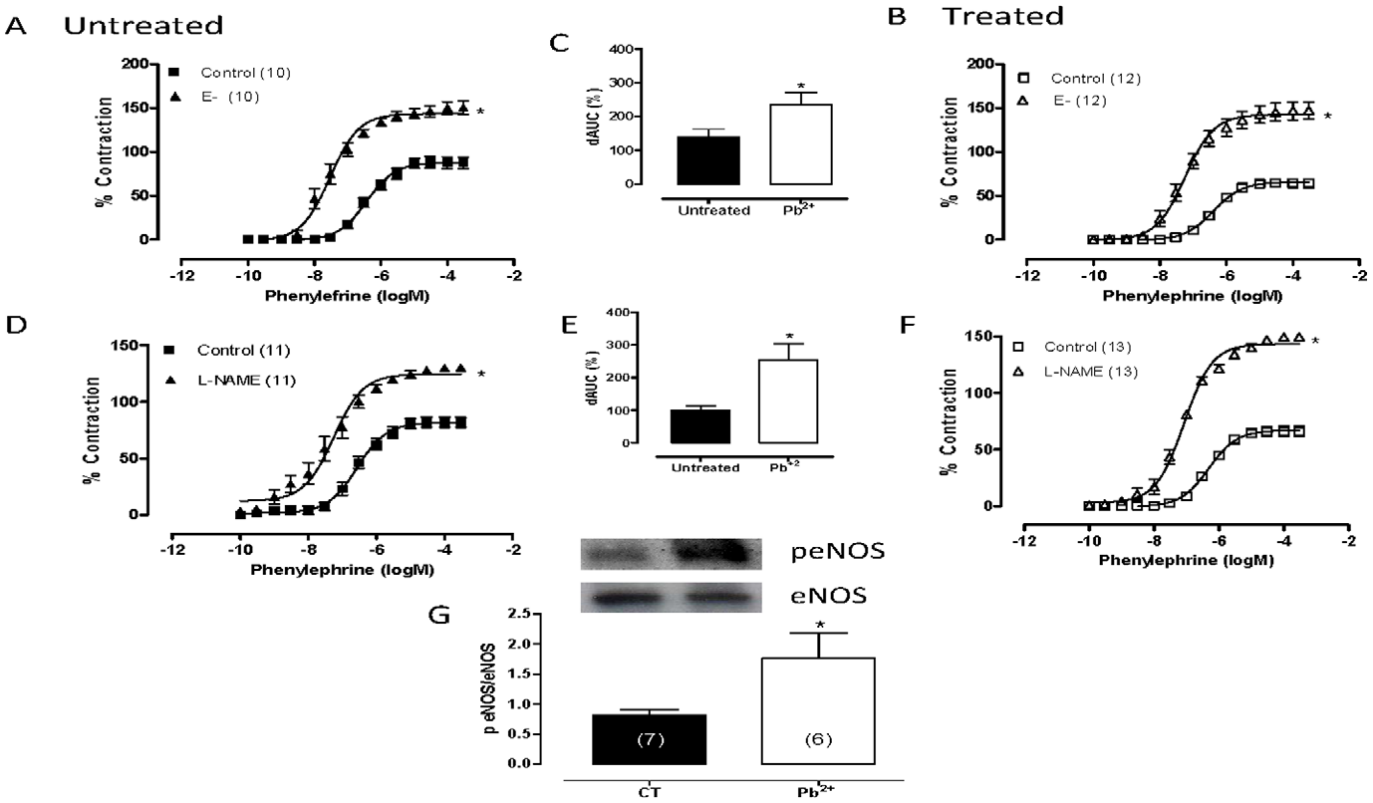
The effects of endothelium removal (E^−^) (A, B) and *N*^G^-nitro-L-arginine methyl ester (L-NAME, 100 µM) (D, E) on the concentration-response curve for phenylephrine treatment in aortic rings from untreated (CT) and lead-treated rats (Pb^+2^). The inset shows differences in area under the concentration-response curves (dAUC) in endothelium–denuded and intact segments (C) and in the presence and absence of L-NAME (F). Densitometry analyses of western blots for endothelial nitric oxide synthase (eNOS) and phosphorylated endothelial nitric oxide synthase (p-eNOS) protein expression in aortas from untreated (CT) and lead-treated rats (Pb^+2^) (G). Representative blots are also shown. *P<0.05 by Student's *t-*test. Number of animals used is indicated in parentheses.

Results are expressed as mean ± SEM of the number of animals shown in Fig. 2; R_max_, maximal effect (expressed as a percentage of the maximal response induced by 75 mM KCl); pD_2_, −log one-half R_max_; Control; E^−^, endothelium removal; _L_-NAME, *N*^G^-nitro-L-arginine methyl ester; indomethacin. P<0.05 *vs.* untreated control rats (^#^) and lead-treated control rats (*).

The concentration-dependent relaxation induced by ACh did not change in the treated group compared to the untreated group (*R*_max_ untreated: 98.90±0.97, n = 8; *R*_max_ lead-treated: 99.37±0.50, n = 9; pD_2_ untreated: 7.13±0.32, n = 8; pD_2_ lead-treated: 7.58±0.58, n = 9). Similarly, the response induced by NPS also did not change in both groups (*R*_max_ untreated: 100±0.24, n = 8; *R*_max_ lead-treated: 100±0.19, n = 9; pD_2_ untreated: 7.63±0.33, n = 8; pD_2_ lead-treated: 7.43±0.13, n = 9) (Figures 1 B and C).

### Effects of lead treatment on endothelial modulation of vasoconstrictor responses

Both endothelium removal and incubation with the NOS inhibitor L-NAME (100 µM) shifted the concentration-response curves to the left after phenylephrine treatment in aortic segments from either group, and this shift was greater in preparations from lead-treated than untreated rats, as shown by the dAUC values (Figures 2 A, B, C, D, E and F; Table 1). Lead treatment did not modify eNOS protein expression in the aorta, although it increased eNOS phosphorylation at Ser^1177^ (Figure 2 G).

**Figure 3:**
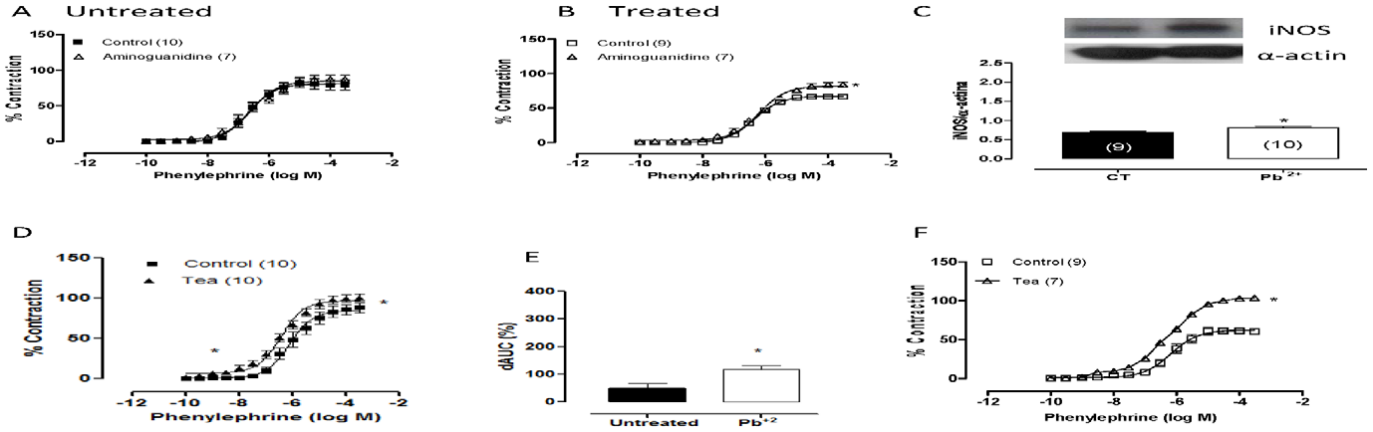
The effects of aminoguanidine (50 µM) (A, B) and TEA (2 mM) (D, E) on the concentration-response curves to phenylephrine in endothelium-intact aortic segments from untreated (CT) and lead-treated rats (Pb^+2^). The inset shows differences in dAUC in the presence and absence of TEA (F). Densitometry analyses of western blots for inducible nitric oxide synthase (iNOS) protein expression in aortas from untreated (CT) and lead-treated rats (Pb^+2^) (C). Representative blots are shown. *P<0.05 by Student's *t-*test. Number of animals used is indicated in parentheses.

Aminoguanidine (50 µM), a nonselective iNOS inhibitor, increased the vasoconstrictor response induced by phenylephrine in aortas from lead-treated rats, but it did not modify the responses to phenylephrine in aortas from control rats (Figures 3 A and B, Table 2). In addition, the protein expression of iNOS increased after lead treatment (Figure 3 C).

**Table 2:** Effects of aminoguanidine, TEA, losartan and enalapril on the vascular responses to phenylephrine (R_max_ and pD_2_) in aortas from untreated and lead-treated rats.

	Untreated		Lead treated	
	Rmax	pD2	Rmax	pD2
**Control**	82.06±6,48	6.02±0,15	60.35±5,99^#^	6.14±0.08
**Aminoguanidine**	86.93±5.56	6.36±0,19	83.71±2,57*	6.23±0.08
**TEA**	100.04±3.70^#^	6.50±0,11^#^	98.29±5,30*	6.30±0,08
**Enalapril**	79.12±2.69	6.21±0.13	48.93±2.53*	6.20±0.13
**Losartan**	79.77±4.12	6.19±0.05	35.13±5.89*	5.98±0.12

Results are expressed as mean ± SEM of the number of animals shown in Figs. 3 and 5 ; R_max_, maximal effect (expressed as a percentage of the maximal response induced by 75 mM KCl); pD_2_, −log one-half R_max_; AG; aminoguanidine, TEA; tetraethylammonium, losartan, enalapril. P<0.05 *vs.* untreated control rats (^#^) and lead-treated control rats (*).

Nitric oxide can open K^+^ channels [35] and contribute to a reduction in contractile responses induced by phenylephrine in aortas from lead-treated rats. TEA (2 mM), a K^+^ channel blocker, potentiated the vasoconstrictor response induced by phenylephrine in aortic segments from either group, but these effects were greater in preparations from lead-treated than untreated rats, as shown by the dAUC values (Figures 3 D, E and F; Table 2).

The cyclooxygenase inhibitor, indomethacin (10 µM), was used to investigate the putative role of prostanoids on the decreased response to phenylephrine in lead-treated rats. Indomethacin did not alter the R_max_ and pD_2_ values after phenylephrine treatment in aortic segments from both groups (Table 1).

### Effects of lead treatment on renin-angiotensin system

**Figure 4:**
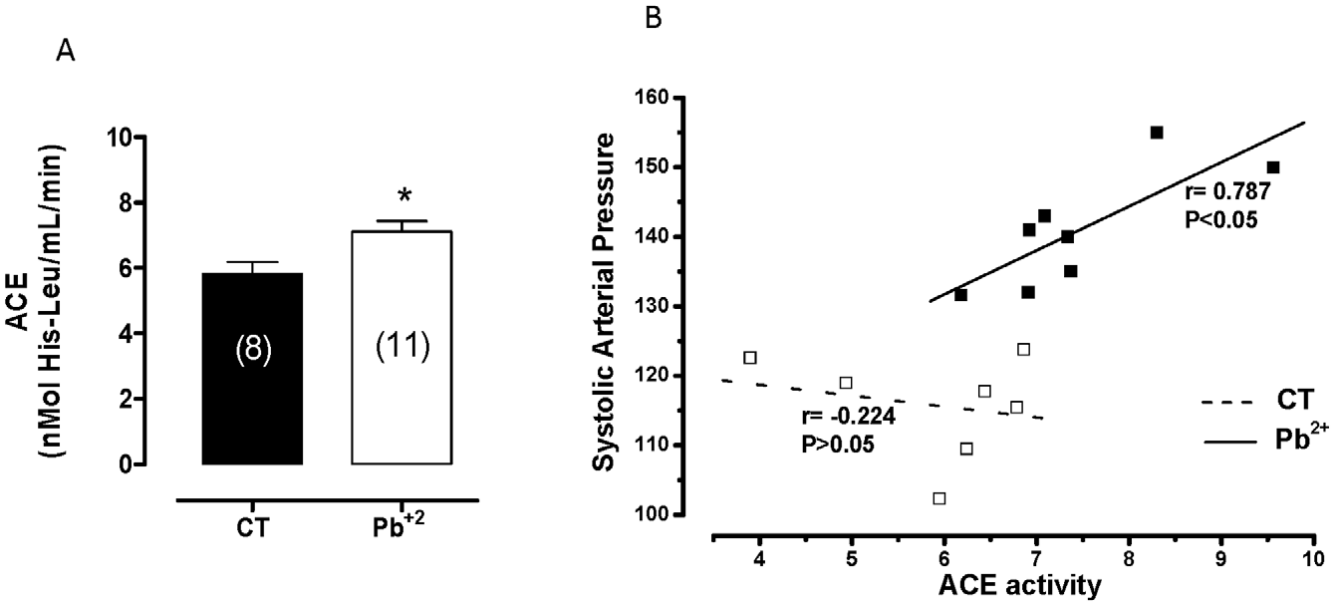
Angiotensin converting enzyme (ACE) activity (nmol His-Leu/min) in plasma; correlation between systolic arterial blood pressure and ACE activity (nmol His-Leu/min) in plasma of control and lead-treated rats (r = 0.787, P<0.05). *P<0.05 by Student's *t-*test. Number of animals used is indicated in parentheses.

To investigate the involvement of the plasma renin-angiotensin system in potentiating the effects of lead exposure, angiotensin converting enzyme activity (ACE) was evaluated. Lead treatment increased plasma ACE activity (Figure 4 A). There was a significant correlation between systolic arterial blood pressure and ACE activity in the plasma of lead-treated rats (r = 0.787, P<0.05; Figure 4 B). These results reinforce the hypothesis that the renin-angiotensin system is involved in the rise in arterial blood pressure in lead-treated rats.

**Figure 5:**
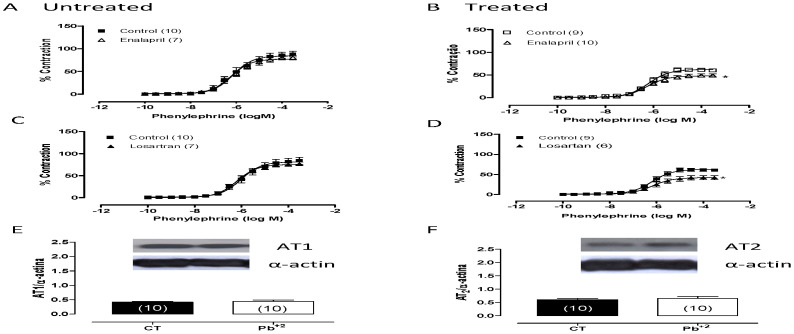
Effect of losartan (10 µM) (A, B) and enalapril (10 µM) (C, D) on the concentration-response curves to phenylephrine in endothelium-intact aortic segments from untreated (CT) and lead-treated rats (Pb^+2^). Densitometry analyses of the western blots for receptors AT_1_ (E) and AT_2_ (F) in aortas from untreated (CT) and lead-treated rats (Pb^+2^). Representative blots are also shown.*P<0.05 by Student's *t-*test. Number of animals used is indicated in parentheses.

To investigate if the local renin-angiotensin system was involved in the alterations of vascular reactivity after phenylephrine treatment induced by lead, ACE and AT_1_ receptors were blocked with enalapril (10 µM) and losartan (10 µM), respectively. As show in Figure 5 (A, B, C and D), both drugs reduced the vasoconstrictor response induced by phenylephrine in aortas from lead-treated rats but not in aortas from control rats (Table 2). This finding suggests that lead affects the local renin-angiotensin system; however, western blot analyses revealed similar levels of AT_1_ and AT_2_ protein expression in the aortas from both groups (Figures 5 E and F).

### Effects of lead treatment on Na^+^/K^+^-ATPase activity

**Figure 6:**
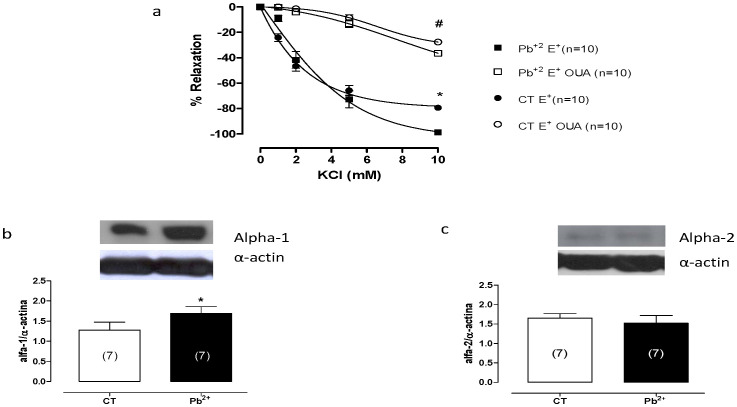
Potassium-induced relaxation in aortic rings from untreated (CT) and lead-treated (Pb^+2^) rats previously incubated in a K^+^-free medium and contracted with phenylephrine before and after incubation with 100 µM ouabain (A). Densitometry analyses of the western blots for the alpha-1 subunit (B) and alpha-2 subunit (C) in aortas from untreated (CT) and lead-treated rats (Pb^+2^). Representative blots are also shown. *P<0.05 (CT *vs.* Pb^+2^) by Student's *t-*test or two-way ANOVA followed by a Bonferroni test. ^#^P<0.05 (CT OUA *vs.* Pb^+2^ OUA) by two-way ANOVA followed by a Bonferroni test. Number of animals used is indicated in parentheses.

We hypothesized that an increase in the Na^+^/K^+^-ATPase activity might be causing the reduction in vascular reactivity after phenylephrine treatment in the aortas from lead-treated rats. The activity of the sodium pump, which was evaluated by the potassium-induced relaxation curves before 100 µM ouabain incubation, increased in aortic rings from treated rats (Figure 6). Preincubation of intact segments with ouabain (100 µM) for 30 min in K^+^-free medium induced an increase in vascular tone in the aortas from both groups, but the increase was smaller in the last concentration of segments from lead-treated rats (Figure 6 A). In addition to this finding, the protein expression of the Na^+^/K^+^-ATPase alpha-1 subunit increased after lead treatment (Figure 6 B). However, the protein expression of the alpha-2 subunit of Na^+^/K^+^-ATPase was similar in the aortas from untreated and lead-treated rats (Figure 6 C).

## Discussion

The major findings from this study indicate that lead treatment used to attain a blood lead content of 9.98 µg/dL is sufficient to increase systolic arterial blood pressure and to decrease the contractile responses induced by phenylephrine in the rat aorta. This blood lead content is much lower than the reference value (60 µg/dL) considered to be the upper limit of exposure in people exposed to lead in their occupation [3], [27], [28]. We found that elevated systolic arterial blood pressure is caused by increased plasma ACE activity. The decrease in the contractile responses after phenylephrine treatment in aortic rings from lead-treated rats is probably due to the increase in negative endothelial modulation. These responses might constitute a counterregulatory mechanism that acts to oppose the increase in blood pressure produced by lead.

The Centers for Disease Control and Prevention (CDC) considers blood lead concentrations greater than or equal 10 µg/dL excessive for infants and children [36]. Blood lead concentrations greater than or equal to 30 µg/dL are considered elevated in adults; however, the Agency for Toxic Substances and Disease Registry (ATSDR) recommends limiting blood lead concentrations to 60 µg/dL in adults whose jobs expose them to lead [3], [27], [28]. It has been reported that adults who experience occupational lead exposure have an increase in blood lead concentration (typically concentrations range from 30 to 60 µg/dL) [6], [37], [9]. In South Korea, individuals who experience occupational lead exposure have blood lead concentrations of approximately 31.4 µg/dL [9]. That study indicated that lead exposure acts continuously on systolic blood pressure, and reductions in exposure may contribute to a decrease in blood pressure. Several studies have supported the association between high blood lead levels and hypertension [38], [8], [39]. In fact, previous reports have suggested that chronic lead exposure at low levels might contribute to hypertension. These studies showed the blood lead concentrations in treated rats were between 31.8 µg/dL and 58.7 µg/dL [29], [30], [13]. In the present study, we treated rats with a low dose of lead for seven days and attained a blood lead concentration of 9.98 µg/dL. This concentration is lower than the levels observed in individuals with occupational lead exposure; nevertheless, this low concentration of lead increased systolic blood pressure in the treated rats.

Lead may induce hypertension via multiple mechanisms, but the pathogenesis is not entirely understood in the initial stages of exposure. Previous studies have shown that lead increased the synthesis and/or release of renin from the juxtaglomerular apparatus [15], the production of aldosterone [40] and the activity of plasma ACE [41], [11]. In our study, increased systolic arterial pressure was accompanied by elevation of plasma ACE activity in lead-treated rats; furthermore, there was a significant correlation between systolic arterial blood pressure and ACE activity in the plasma of lead-treated rats. Previous reports have shown that elevation in systolic blood pressure after 2 weeks of lead treatment (100 ppm lead acetate in drinking water) was accompanied by an increase in plasma ACE activity [19]. These findings suggest that ACE activity might play a role in the initial stages of lead-induced hypertension.

Previous studies have shown that alterations in vascular tone are possibly involved in lead-induced hypertension [30], [42]; however, at low concentrations and in the initial stages of lead exposure, changes in vascular reactivity have not yet been described. Several reports have shown that chronic exposure to lead at low concentrations induces vasoconstriction in the aorta [24], [25], [13]. Chronic exposure to lead (100 ppm for 10 months) also decreased the contractile response induced by 5-HT in the aortas of lead-treated rats [26]. These effects might be mediated by increased production of reactive oxygen species [42], vasoconstrictor prostanoids of the cyclooxygenase pathway [13] and pathomorphological changes in the vessels [26].

In this study, we observed a reduction in the reactivity to phenylephrine in aortic rings after seven days of lead exposure. The reduction in vascular reactivity to phenylephrine was accompanied by a concomitant increase in the endothelial modulation of such responses. To investigate NO modulation, the NOS inhibitor, L-NAME, was used. We observed that L-NAME increased the reactivity to phenylephrine in both experimental groups; however, the magnitude of the effect of L-NAME was higher in lead-treated rats. These results suggest that lead increases NO bioavailability, thereby reducing reactivity to phenylephrine in the aortic rings. In support of these data, we also observed that a seven-day lead exposure increased eNOS phosphorylation at Ser^1177^. This specific residue is the major regulator of NO production [43], [44]. Other investigators have also indicated that high concentrations of lead increases the NO bioavailability in rat tail arteries [45]; however, Karimi et al. [24] showed that treatment with 100 ppm lead acetate for 28 days reduces NO bioavailability in rat aortas. This study was performed with chronic lead treatment, whereas our study was performed in the initial stages of lead exposure. The results concerning the effects of lead on NO production are controversial. Vaziri and Ding et al. [46] showed that incubation with 1 ppm lead acetate for 24 hours increases NO production in human coronary artery endothelial cells; however, other reports have shown that treatment with lead acetate (100 ppm in drinking water) for 12 weeks decreases [47], [20], [21], [48] urine NO concentration. These contradictory results are probably due to differences in the timing and the form of lead treatment.

To investigate whether iNOS has a putative role mediating the effects of lead treatment, aminoguanidine, an iNOS blocker, was used. Aminoguanidine increased the effects of lead on the contractile response to phenylephrine. Similarly, we found that that lead exposure increased iNOS protein expression. These results suggest that NO from iNOS also mediates the reduced reactivity to phenylephrine induced by lead. Several reports have also suggested that chronic exposure to lead increases the expression of iNOS in the aorta [21], [48], heart [48] and kidney [29], [48]. Although we demonstrated an increase in NO bioavailability induced by lead, the relaxations induced by acetylcholine and sodium nitroprusside were not altered after seven days of lead exposure. These results indicate that after seven days, lead does not seem to modify the release of NO stimulated by acetylcholine or alter NO signaling pathways. In agreement with our findings, Grizzo et al. [13] showed that lead did not change the induced acetylcholine- and sodium nitroprusside-induced relaxations in lead-treated rats.

The increased NO could open K^+^ channels and contribute to increased negative modulation of the phenylephrine contraction. We demonstrated that TEA, a K^+^ channel blocker, potentiated the response to phenylephrine in aortic segments from either group, but these effects were greater in preparations from lead-treated than from untreated rats, as shown by dAUC values. These findings suggest that NO might be activating K^+^ channels in vascular smooth muscle cells, as previously reported [35], [49].

Prostacyclin is another endothelium-derived vasodilator that might be involved in negative endothelial modulation of aortic rings from lead-treated rats. It has been shown that lead exposure increases the activity of the cyclooxygenase pathways in aortic rings [24], [13] and in the tail vascular bed of rats [45]. In the present study, indomethacin did not modify vascular reactivity to phenylephrine in untreated and lead-treated rats. These findings suggest that the cyclooxygenase pathway, at least in these initial stages of exposure, is not involved in the decreased vascular reactivity in aortas from lead-treated rats.

Seven days of lead exposure increased arterial systolic pressure, even though vascular reactivity to phenylephrine decreased due to an increased NO bioavailability in aortic rings. A previous study showed that local angiotensin II stimulates the production of NO in aortic endothelial cells [50]; therefore, we investigated whether the local renin-angiotensin system might be involved in the alterations of vascular reactivity to phenylephrine induced by lead. We showed that losartan and enalapril decreased the contractile response to phenylephrine in aortic rings from lead-treated rats, which is in agreement with the increased plasma ACE activity found in these rats. Despite the involvement of the local renin-angiotensin system in this experimental model, which could induce vasoconstriction, the vasodilatory effects of NO were more significant and contributed to a reduction in the vascular reactivity to phenylephrine. Western blot analyses showed similar levels of AT_1_ and AT_2_ protein expression in the aortas from both groups. These findings suggest that seven days of lead treatment was not sufficient to produce alterations in the expression of these receptors, even under conditions of increased plasma ACE activity.

In this study, we showed that lead treatment induced the release of endothelium-derived factors such as nitric oxide and angiotensin II; however, both nitric oxide and angiotensin II stimulate Na^+^/K^+^-ATPase activity, which might reduce vascular tone [51], [52], [53]. Therefore, we investigated the effects of lead treatment on Na^+^/K^+^-ATPase functional activity, and we demonstrated that lead increases Na^+^/K^+^-ATPase functional activity. We analyzed this activity further by evaluating the expression of the alpha-1 and alpha-2 subunits of the Na^+^/K^+^-ATPase. Western blot analyses showed that the seven-day lead exposure increased the protein expression of the Na^+^/K^+^-ATPase alpha-1 subunit; however, the Na^+^/K^+^-ATPase alpha-2 subunit was present in similar levels in aortas from untreated and lead-treated rats. Thus, the increased NO bioavailability and angiotensin II production induced by lead could be causing increased Na^+^/K^+^-ATPase functional activity.

In summary, seven day of lead treatment increased systolic arterial blood pressure and reduced vascular reactivity to phenylephrine in aortic segments from rats. The increased plasma ACE activity could contribute to elevated systolic blood pressure in lead-treated rats. In contrast, the decreased vascular reactivity due to increased NO bioavailability might constitute a counterregulatory mechanism against the elevated systolic arterial pressure observed in these animals, at least in the initial stages of lead exposure. Lead treatment also increased p-eNOS and iNOS protein expression. Presumably, the increased NO could open K^+^ channels and contribute to increased negative modulation of the phenylephrine contraction. In addition, both angiotensin II and NO bioavailability can stimulate Na^+^/K^+^-ATPase functional activity by decreasing vascular reactivity. In conclusion, our results show that vascular changes and increased systolic blood pressure occur in the initial stages of low-concentration lead exposure, and thus, lead can be considered an important risk factor for cardiovascular disease.
